# Mental health and sleep quality among patients with asthma and COPD

**DOI:** 10.3389/fmed.2023.1181742

**Published:** 2023-05-09

**Authors:** Yousef Saad Aldabayan

**Affiliations:** Department of Respiratory Care, King Faisal University, Al Asha, Saudi Arabia

**Keywords:** chronic obstructive pulmonary disease (COPD), asthma, sleep disturbance, anxiety, depression

## Abstract

This study aims to compare the mental health of patients with asthma and COPD in terms of anxiety, depression, and sleep quality and to examine the factors that predict sleep disturbance, anxiety, and depressive symptoms.

**Methods:**

This quantitative cross-sectional study employed convenience sampling to enroll 200 patients with asthma and 190 patients with COPD. Data were gathered using a standardized self-administered questionnaire that contained sections on patients’ characteristics, the Sleep Quality, Anxiety, and Depression.

**Results:**

The prevalence of poor sleep quality was 17.5 and 32.6% among asthmatic and COPD patients, respectively. The incidence of anxiety and depression was 38 and 49.5% among the patients with asthma, respectively. Their prevalence in patients with COPD was 48.9 and 34.7%, respectively. The multivariate regression analysis showed that marital status (married), BMI, education level (pre-university level), presence of comorbid illness, and depression were significant predictors of PSQI in asthmatic patients. Moreover, age, gender (male), marital status (married), education level (pre-university level), depression, and anxiety were significant predictors of PSQI among COPD participants. According to this study, COPD, and asthma pose serious health risks, including reduced sleep quality, anxiety, and depression.

## 1. Introduction

Chronic respiratory disorders account for 10% (4.1 million) of non-communicable disease mortality, and 74% (40 million) of all fatalities (1). Two of the most prevalent chronic lung diseases worldwide are asthma and chronic obstructive pulmonary disease (COPD) (2). Asthma and COPD are also the most prevalent serious illnesses in Saudi Arabia, and local reports indicate that these conditions are becoming more prominent (3). As the industrialization and modernity of the last ten years have increased, so has the prevalence of asthma. According to the World Health Organization (WHO), there were over 383,000 asthma-related deaths in 2015, affecting 235 million people worldwide (4). The Global Initiative for Chronic Obstructive Lung Disease (GOLD) asserts that COPD is a prevalent, curable condition (5). Over the following 10 years, COPD is anticipated to overtake all other significant causes of mortality as the third leading cause of death worldwide (5). In 2019, there were 3.9 million mortalities and an estimated 455 million cases of COPD (6).

More than 2 million Saudis suffer from asthma, and most of them have uncontrolled asthma, which negatively influences their quality of life. According to the Saudi Arabian (SA) Ministry of Health, 15–25% of people in SA have asthma (1). Despite improvements in modern medicine, 40–70% of people still have uncontrolled asthma (7). In addition, an epidemiological study that sought to determine the prevalence and incidence of COPD in Saudi Arabia from 1990 to 2019 found that the number of instances of the disease was rising: from 1,381.26 (1,285.35–1,484.96) cases per 100,000 people in 1990 to 2,053.04 cases per 100,000 people in 2016 (1918.06–2194.29) (8).

Patients with asthma and COPD experience significant impairments in their physical and mental health, which considerably impair their performance in day-to-day activities and social interactions and raise their risk of morbidity and mortality (9). In addition, asthma, and COPD are both prevalent, not fully curable diseases typically characterized by a slowly progressing and persistent airflow restriction that may cause sleep disturbances (1). The symptoms of sleep disorders that can interfere with daytime activities include the inability to maintain a high level of sleep quality or problems with sleep duration. Patients with asthma frequently complain of sleep disruptions, including early morning awakenings and trouble falling or staying asleep (3, 4, 7, 8).

Previous researches suggest a relationship between sleep quality and mental health among individuals with COPD and asthma. Sleep disorders are correlated with the severity of asthma, and sleep quality in COPD patients is an essential determinant of quality of life (9, 10). Centers for Diseases Control and prevention reported that mental and physical health are equally important components of overall health, and mental health includes emotional, psychological, and social well-being (11). In addition, there is a close association between mental health and respiratory diseases. For instance, patients with COPD and asthma are more likely to experience anxiety and depression (12, 13).

Asthma and depression are linked, and depression has been linked to sleep disorders, poor sleep quality, and lower quality of life (14). Compared to the general population, patients with asthma and COPD have approximately three times the prevalence of sleep disruption, anxiety, and depression. Therefore, asthma, and COPD treatments should include early diagnosis and multi-modal therapy for sleep disruption, anxiety, and depression (15). However, to date, few studies have investigated the predictors of asthma and COPD. This study compared the mental health of patients with asthma and COPD in terms of anxiety, depression, and sleep quality. In addition, the current research examines the factors that predict sleep disturbance, anxiety, and depressive symptoms in individuals with asthma and COPD.

## 2. Materials and methods

### 2.1. Setting and design

The study was carried out in primary healthcare facilities with smoking and respiratory clinics in Alhufof, in the eastern Saudi Arabian province of Al-Ahsa. To achieve the study’s objectives, a quantitative cross sectional research design was used.

### 2.2. Participants

A convenience sampling of 200 asthma patients and 190 COPD patients was conducted. The eligible criteria were: both gender, age > 18 years, stable health status, verbal, and cognitive ability, absence of mental illnesses, and agreement to participation. Clinical history, physical examination, and spirometry were used in our research setting to diagnose asthma following the Global Initiative for Asthma (GINA) guidelines. In addition, according to the Global Initiative for Chronic Obstructive Lung Disease (GOLD) recommendations, spirometry is the gold standard for diagnosing COPD, therefore, only those patients diagnosed by their physician based on their spirometry results were included. Exclusion criteria included patients with malignancy, acute or exacerbations of respiratory conditions, and patients whose diagnoses had not been verified were excluded. In addition, all patients had to be clinically stable, meaning they experienced no exacerbation in the four weeks before recruitment.

### 2.3. Data collection

Data were gathered using a standardized self-administered questionnaire. It contained two standardized tools and a section on patient characteristics, such as age, gender, marital status, degree of education, BMI, respiratory disease in the family, smoking history, and comorbid illness.

The Sleep Quality Pittsburgh Sleep Quality Index (PSQI) was used to evaluate subjective sleep problems over a one-month period (16). This is a standardized questionnaire, and previous research has evaluated and confirmed its validity and reliability for the Arab community (17, 18). Therefore, the Arabic version was chosen for our study (19). The PSQI is a 19-item self-rating scale that assesses the perceived quality of sleep for a previous month. It includes seven domain ratings for subjective sleep quality, sleep latency, sleep duration, habitual sleep efficiency, sleep disruptions, the use of sleeping medications, and daytime dysfunction. A higher score denotes poorer sleep quality, and each item is weighted on a 0–3 scale. Global ratings are based on a scale of 0 to 21, with a score of five or more indicating bad sleep and a score of five or less indicating good sleep (16).

The Hospital Anxiety and Depression Score (HADS), a self-report test used frequently in non-psychiatric settings to assess the two most common expressions of distress—anxiety and depression—was the second tool. The HADS consists of seven questions each for anxiety and depression. Each item is scored on a four-point scale, with 0 denoting “not present” and 3 denoting a “considerable event.” The total score on these two subscales ranges from 0 to 21. The threshold score is eight or higher. A score of eight or more on the depression and anxiety subscales is regarded as symptomatic or an atypical condition. The HADS has a specificity of 0.78 and a sensitivity of 0.9 for anxiety and a specificity of 0.79 and a sensitivity of 0.83 for depression (20, 21). The Arabic version was used. Cronbach’s alpha for the HADS depression and anxiety subscales was 0.77 (0.7–0.83) and 0.83 (95% confidence interval: 0.79–0.88), respectively (22).

Five patients participated in a pilot research to evaluate the tool’s visibility, filling speed, and usefulness. Each participant was examined by the researchers in light of the above-mentioned inclusion criteria as well as the objectives and aims of the study. Those who agreed to participate were then asked to sign an informed consent form and complete the questionnaire. The questionnaire was completed in 10 to 15 min by each participant. The data collection took approximately 6 months, between October 2021 and March 2022.

### 2.4. Ethical considerations

The Research and Ethical Committee, Ministry of Health, authorized the research proposal (approval number IRB KFHH no. H-05-HS-065). After outlining the purpose of the study, the patients gave their informed consent. Participation in this study was entirely voluntary. The patient’s confidentiality, privacy, anonymity, and right to withdraw from the study at any time were guaranteed. The Declaration of Helsinki (23) was observed, and ethical guidelines were followed when conducting the study.

### 2.5. Statistical analysis

Version 28 of the SPSS (Statistical Package for the Social Sciences) software was used to collect and analyze the data. The results of the descriptive statistics were given as a number (%) or mean (SD) for the categorical and continuous variables, respectively. The data’s normality was analyzed graphically. The PSQI and depression and anxiety were correlated using Pearson’s correlation coefficient. In order to determine which patient features were highly predictive of insufficient sleep, sadness, and anxiety, numerous linear regression analyses were conducted. With a 95% confidence interval, a *p* value of 0.05 was deemed significant. To account for all factors, we also performed multivariate linear regressions.

## 3. Results

Table 1 shows that 200 patients with asthma and 190 patients with COPD were enrolled in the current study. The mean (SD) ages of the patients with asthma and patients with COPD were 36.23 ± 10.19 years and 47.06 ± 10.91, respectively. Most patients in the asthma group (77%) were female (58.5%), while 61.1% of patients in the COPD group were male. The majority of the study participants had no history of previous hospital admissions.

**Table 1:** Distribution of characteristics of patients with asthma (*N* = 200) and COPD (*N* = 190).

Characteristics	Patient with asthma	Patient with COPD
	*N* (%) Mean SD	*N* (%) Mean SD
Age	36.23 ± 10.19	47.06 ± 10.91
Gender		
Male	46 (23.0)	116 (61.1)
Female	154 (77.0)	74 (38.9)
Marital status		
Single	86 (43.0)	79 (41.6)
Married	114 (57.0)	111 (58.4)
Education level		
Pre-university	153 (76.5)	131 (68.9)
University level	47 (23.5)	59 (31.1)
BMI		
Normal	59 (29.5)	4 (2.1)
Overweight	138 (69.0)	46 (24.2)
Obese	3 (1.5)	140 (73.7)
Family history of respiratory diseases		
Yes	31 (15.5)	17 (8.9)
No	169 (84.5)	173 (91.1)
Smoking (lifetime)		
Yes	24 (12.0)	141 (74.2)
No	176 (88.0)	49 (25.8)
Previous hospital admission for exacerbation		
Yes	48 (24.0)	15 (7.9)
No	152 (76.0)	175 (92.1)
Comorbid illness		
None	153 (76.5)	141 (74.2)
Anemia	14 (7.0)	9 (4.7)
Hypertension	22 (11.0)	24 (12.6)
Diabetes Mellitus	8 (4.0)	16 (8.4)
Dysrhythmia	3 (1.5)	0 (0)

Table 2 shows the frequency of the PSQI elements. The frequency of poor sleep quality was 17.5 and 32.6% among the asthma and COPD groups, respectively. More than half of the participants reported sleeping fairly well. About 34.5% in the asthma group had sleep latency of 16–30 min, while 36.3% in the COPD group had 31–60 min of sleep latency. About 59.5% of the asthma group and 71.1% of the COPD group informed sleep disturbances at least once per week. Furthermore, more than 50% of study participants claimed to have daytime dysfunction, and the majority of patients in both asthma and COPD groups did not use sleep medications.

**Table 2:** Frequency of PSQI elements of participants with asthma (*N* = 200) and COPD (*N* = 190).

Elements	Asthma *N* (%)	COPD *N* (%)
Subjective sleep quality		
Very good	46 (23.0)	54 (28.4)
Fairly good	110 (55.0)	122 (64.2)
Fairly bad	29 (14.5)	14 (7.4)
Very bad	15 (7.5)	0 (0)
Sleep latency		
<15 min	24 (12.0)	22 (11.6)
16–30 min	69 (34.5)	59 (31.1)
31–60 min	55 (27.5)	69 (36.3)
>60 min	52 (26.0)	40 (21.1)
Sleep duration		
>7 h	55 (27.5)	41 (21.6)
6–7 h	60 (30.0)	53 (27.9)
5–6 h	34 (17.0)	58 (30.5)
<5 h	51 (25.5)	38 (20.0)
Sleep efficiency		
>85%	129 (64.5)	99 (52.1)
75–84%	35 (17.5)	51 (26.8)
65–74%	21 (10.5)	17 (8.9)
<65%	15 (7.5)	23 (12.1)
Sleep disturbance		
Not in the previous month	0 (0)	0 (0)
Less than once a week	119 (59.5)	135 (71.1)
Once or twice a week	81 (40.5)	55 (28.9)
Three or more times a week	0 (0)	0 (0)
Use of sleep medication		
Not during past month	140 (70.0)	157 (82.6)
Less than once per week	34 (17.0)	18 (9.5)
Once or twice a week	26 (13.0)	8 (4.2)
At least three times every week	0 (0)	7 (3.7)
Daytime dysfunction		
Not during past month	56 (28.0)	107 (56.3)
Less than once a week	54 (27.0)	51 (26.8)
Once or twice a week	81 (40.5)	25 (13.2)
Three or more times a week	9 (4.5)	7 (3.7)
Good Sleep Quality (<4)	165 (82.5)	128 (67.4)
Poor Sleep Quality(>5)	35 (17.5)	62 (32.6)

Table 3 shows the prevalence of depression and anxiety was 49.5 and 38% among patients with asthma, respectively. In COPD patients, the prevalence was 48.9 and 34.7%, respectively.

**Table 3:** Frequency of anxiety and depression among patients with asthma (*N* = 200) and COPD (*N* = 190).

Variable	*N* (%)	
**Depression (Total Score)**	**Asthma**	**COPD**
Normal (0–7)	101 (50.5)	97 (51.1)
Abnormal (8–21)	99 (49.5)	93 (48.9)
**Anxiety (Total Score)**		
Normal (0–7)	124 (62.0)	124 (65.3)
Abnormal (8–21)	76 (38.0)	66 (34.7)

Table 4 shows the factors of poor sleep quality among patients with asthma and COPD.

**Table 4:** Regression analyses of poor sleep quality based on the characteristics of patients with asthma (*N* = 200) and COPD (*N* = 190).

	Asthma				COPD			
Independent variables	Univariate model: *β* (95% CI)	*p*	Multivariate model: *β* (95% CI)	*p*	Univariate model: *β* (95% CI)	*p*	Multivariate model: *β* (95% CI)	*p*
Age	0.009 (0.003, 0.014)	0.001	0.004 (−0.002, 0.010)	0.206	−0.014 (−0.020, −0.008)	<0.001	−0.009 (−0.015, −0.003)	0.002
Gender (Male)	0.083 (−0.043, 0.209)	0.194	0.119 (−0.014, 0.252)	0.080	0.180 (0.044, 316)	0.010	0.271 (0.134, 0.408)	<0.001
Marital status (married)	0.203 (0.099, 0.307)	<0.001	0.110 (−0.004, 0.224)	0.059	0.330 (0.201, 0.458)	<0.001	0.267 (155, 0.379)	<0.001
BMl	−0.060 (−0.171, 0.050)	0.281	−0.111 (−0.218, −0.004)	0.042	−0.013 (−0.149, 0.123)	0.847	0.096 (−0.020, 0.211)	0.106
Family history of the disease (no)	−0.016 (−0.163, 0.131)	0.828	−0.120 (−0.281, 0.040)	0.142	−0.358 (−0.589, −0.128)	0.002	−0.159 (−0.387, 0.068)	0.169
Smoking (lifetime) (no)	−0.009 (−0.173, 0.154)	0.909	0.014 (−0.146, 0.175)	0.860	0.082 (−0.072, 0.236)	0.293	−0.118 (−0.259, 0.024)	0.103
Education level (pre-university level)	−0.188 (−0.311, −0.066)	0.003	−0.278 (−0.403, −0.153)	<0.001	−0.166 (−0.310, −0.022)	0.024	−0.161 (−0.305, −0.016)	0.029
Previous hospital admission (no)	−0.066 (−0.190, 0.059)	0.298	−0.212 (−0.349, −0.075)	0.003	−0.065 (−0.315, 0.185)	0.610	−0.168 (−0.457, 0.121)	0.253
Comorbid illness (yes)	0.087 (0.032, 0.142)	0.002	0.104 (0.046, 0.162)	<0.001	−0.061 (−0.128, 0.005)	0.071	−0.072 (−0.132, −0.013)	0.018
Dyspnea last month (no)	−0.048 (−0.154, 0.059)	0.379	0.080 (−0.033, 0.193)	0.166	−0.233 (−0.364, −0.102)	0.001	−0.093 (−0.258, 0.072)	0.267
Depression (yes)	0.247 (0.146, 0.347)	<0.001	0.175 (0.068, 0.282)	0.001	−0.035 (−0.170, 0.100)	0.611	−0.378 (−0.527, −0.228)	<0.001
Anxiety (yes)	0.070 (−0.039, 0.179)	0.208	0.053 (−0.058, 0.164)	0.350	0.337 (0.204, 0.471)	<0.001	0.433 (0.269, 0.598)	<0.001

Model *R*-square of participants with asthma = 0.299, value of *p* < 0.001. Model *R*-square of participants with COPD = 0.471, value of *p* < 0.001.

In patients with asthma, the univariate linear regression model showed that the predictors of poor sleep quality were age, marital status (married), education level (pre-university level), presence of comorbid illness, and depression. These were independently associated with worse sleep quality. In the multivariate regression model, marital status (married), BMI, education level (pre-university level), presence of comorbid illness, and depression were the significant predictors of PSQI.

In patients with COPD, the univariate linear regression model showed that the predictors for poor sleep quality were age, gender (male), marital status (married), family history of the disease (no), education level (pre-university level), presence of comorbid illness, and anxiety independently associated with worse sleep quality. In the multivariate regression model, age, gender (male), marital status (married), education level (pre-university level), depression, and anxiety were significant predictors of PSQI for COPD patients, as shown in Table 4.

Table 5 displays that age, gender, marital status, education level (pre university level), and anxiety were all independent predictors of a higher level of depression in patients with asthma. In patients with COPD, BMI, family history of respiratory disease, smoking, pre-university education level, prior hospital admission, prior dyspnea, and anxiety were the factors that independently predicted an escalating level of depression.

**Table 5:** Regression analysis of depression and anxiety scores based on the characteristics of patients with asthma (*N* = 200) and COPD (*N* = 190).

	Depression							
	Asthma				COPD			
Independent variables	Univariate model: *β* (95% CI)	*p*	Multivariate model: *β* (95% CI)	*p*	Univariate model: *β* (95% CI)	*p*	Multivariate model: *β* (95% CI)	*p*
Age	0.018 (0.011, 0.024)	<0.001	0.018 (0.010, 0.026)	<0.001	0.000 (−0.006, 0.007)	0.930	0.003 (−0.002, 0.009)	0.217
Gender (Male)	−0.232 (−0.396, −0.069)	0.006	−0.245 (−0.42-, −0.070)	0.006	−0.071 (−0.218, 0.076)	0.340	−0.097 (−0.231, 0.037)	0.156
Marital status (Married)	0.297 (0.162, 0.432)	<0.001	0.101 (−0.052, 0.254)	0.195	0.079 (−0.066, 0.225)	0.282	−0.015 (−0.126, 0.096)	0.788
BMl	−0.028 (−0.173, 0.118)	0.708	−0.054 (−0.198, 0.090)	0.457	0.031 (−0.114, 0.176)	0.677	0.116 (0.003, 0.229)	0.045
Family history of the respiratory disease (Yes)	0.051 (−0.142, 0.245)	0.601	0.012 (−0.204, 0.229)	0.910	0.085 (−0.166, 0.337)	0.504	0.304 (0.084, 0.524)	0.007
Smoking(lifetime) (No)	−0.006 (−0.221, 0.210)	0.959	0.163 (−0.052, 0.378)	0.137	−0.110 (−0.273, 0.054)	0.188	−0.138 (−0.276, 0.001)	0.051
Education level (Pre-university level)	−0.119 (−0.283, 0.046)	0.156	−0.211 (−0.377, −0.046)	0.013	−0.267 (−0.418, −0.117)	0.001	−0.317 (−0.452, −0.182)	<0.001
Previous hospital admission (No)	−0.007 (−0.171, 0.157)	0.937	−0.083 (−0.267, 0.101)	0.376	−0.410 (−0.670, −0.149)	0.002	−0.559 (−0.833, −0.286)	<0.001
Comorbid illness (Yes)	0.025 (−0.049, 0.099)	0.505	−0.007 (−0.085, 0.071)	0.854	0.071 (0.000, 0.142)	0.049	0.002 (−0.057, 0.061)	0.949
Dyspnea last month (No)	−0.161 (−0.299, −0.022)	0.023	−0.058 (−0.210, 0.094)	0.451	−0.580 (−0.697, −0.462)	<0.001	−0.366 (−0.520, −0.213)	<0.001
Anxiety (Yes)	0.178 (0.036, 0.320)	0.014	0.190 (0.043, 0.337)	0.012	0.620 (0.498, 0.742)	<0.001	0.288 (0.131, 0.445)	<0.001
Anxiety								
Age	0.012 (−0.011, 0.002)	0.215	−0.005 (−0.012, 0.003)	0.221	−0.005 (−0.011, 0.001)	0.129	0.000 (−0.005, 0.006)	0.894
Gender (Male)	−0.099 (−0.260, 0.062)	0.225	−0.042 (−0.214, 0.130)	0.631	−0.038 (−0.178, 0.103)	0.596	−0.259 (−0.379, −0.139)	<0.001
Marital status (Married)	0.075 (−0.062, 0.212)	0.281	−0.016 (−0.165, 0.134)	0.837	0.140 (0.002, 0.277)	0.047	0.046 (−0.058, 0.150)	0.381
BMl	−0.210 (−0.348, −0.072)	0.003	−0.151 (−0.290, −0.012)	0.034	−0.005 (−0.143, 0.133)	0.941	0.001 (−0.105, 0.107)	0.986
Family history of the disease (Yes)	0.259 (0.074, 0.443)	0.006	0.367 (0.367, 0.572)	0.001	−0.071 (−0.311, 0.169)	0.561	−0.200 (−0.405, 0.004)	0.055
Smoking(lifetime) (No)	−0.042 (−0.251, 0.168)	0.695	082 (−0.292, 0.129)	0.445	0.054 (−0.102, 0.211)	0.493	0.137 (0.009, 0.265)	0.037
Education level (Pre-university level)	0.032 (−0.129, 0.192)	0.697	0.152 (−0.008, 0.313)	0.063	−0.332 (−0.472, −0.192)	<0.001	−0.352 (−0.468, −0.237)	<0.001
Previous hospital admission (No)	0.226 (0.070, 0.382)	0.005	0.291 (0.116, 0.466)	0.001	−0.130 (−0.383, 0.124)	0.315	0.203 (−0.052, 0.457)	0.119
Comorbid illness (Yes)	0.108 (0.038, 0.179)	0.003	0.131 (0.057, 0.204)	0.001	0.087 (0.019, 0.154)	012	0.073 (0.019, 0.127)	0.009
Dyspnea last month (No)	−0.122 (−0.257, 0.013)	0.076	−0.342 (−0.482, −0.201)	<0.001	−0.572 (−0.682, −0.463)	<0.001	−0.630 (−0.740, −0.520)	<0.001
Depression (Yes)	0.178 (0.036, 0.320)	0.014	0.190 (0.043, 0.337)	0.012	0.620 (0.498, 0.742)	<0.001	0.288 (0.131, 0.445)	<0.001

For asthma participants: Model *R*-square for depression = 0.267, value of *p* < 0.001, Model *R*-square for anxiety = 0.248, value of *p* < 0.001. For COPD participants: (Model *R*-square for depression = 0.543, value of *p* < 0.001, Model *R*-square for anxiety = 0.554, value of *p* < 0.001).

Moreover, BMI, family history of respiratory disease, previous hospital admission, the presence of comorbidities, dyspnea within the past month, and depression were the factors that independently predicted a higher level of anxiety among asthmatic patients. However, among COPD patients, factors such as male gender, family history of respiratory disease, smoking, pre-university education level, presence of comorbidities, dyspnea within the past month, and depression were associated with higher levels of anxiety.

Table 6 shows that there is correlation between sleep quality, anxiety, and depression among patients with asthma and COPD (*N* = 390).

**Table 6:** Correlation between sleep quality, anxiety, and depression among patients with asthma and COPD (*N* = 390).

Variables		Correlation	*p*-value
Diagnosis: (asthma and COPD)	PSQI	0.175**	0.001
	Depression	0.006	0.913
	Anxiety	0.034	0.504
PSQI	Depression	0.128*	0.012
	Anxiety	226**	<0.001
Depression	Anxiety	0.374**	<0.001

**Correlation is significant at the 0.01 level (2-tailed), *Correlation is significant at the 0.05 level (2-tailed).

## 4. Discussion

A significant sample size of asthma and COPD patients was used in the study to explore the prevalence of mental health problems and poor sleep quality, in Al-Ahsa, Saudi Arabia. Poor sleep, depression, and anxiety were somewhat common in this patient group, and a variety of factors contributed to this frequency. Healthcare providers should therefore be alert to these disorders and address them promptly and effectively.

Research on the prevalence of poor sleep quality in the Saudi population is lacking, despite the fact that the prevalence of poor sleep quality in patients with respiratory illnesses has been extensively studied in the literature. In our study, we found that poor sleep quality has a higher incidence among patients with COPD than among those with asthma (32.6% vs. 17.5%), as measured by the PSQI. Asthmatics had longer sleep duration, efficiency, and latency, whereas COPD patients had better sleep quality, fewer sleep disturbances, and used fewer sleep medications than asthmatics. This is consistent with a study (24) that found that the PSQI scores for COPD were higher than those for asthma. In contrast, our study found that COPD patients had longer sleep latency and used sleep-regulating medications more often. The disparity in the reported prevalence of sleep components is probably due to methodological variations, such as sample size and study design. Our findings also support a study (25) that reported a 35% (*N* = 245) incidence of poor sleep quality in patients with chronic respiratory diseases Furthermore, another study (26) reported that more than half (53% of 1,117) of the participants with COPD reported “poor” sleep quality.

With sleep occupying up to one-third of every adult’s life, addressing sleep is essential to overall health. Sleep disturbances and deficiencies are common in patients with chronic lung diseases and are associated with worse clinical outcomes and poor quality of life (27). The causes of poor sleep quality in patients with asthma and COPD remains unclear. However, a variety of considerations have been proposed. A prior study (28) demonstrated a significant correlation between respiratory problems and insufficient sleep. Patients with asthma and COPD frequently encounter symptoms, such as coughing, shortness of breath, dyspnea, and wheezing. These symptoms have been linked to higher rates of insomnia and daytime sleepiness than people who do not exhibit them. Dyspnea, a defining feature of COPD, secretion buildup along with mucus clogging the airways, poor ventilation and inadequate oxygenation have all been found to significantly impact sleep quality (29, 30).

Patients with chronic lung diseases frequently experience mental health problems as anxiety and depression, which have a serious impact on their health and prognosis. According to a randomized cross-sectional study (31) conducted in Sharurah, Saudi Arabia, 12% of 280 patients evaluated in primary healthcare facilities were diagnosed with depression. In the present study, we found the prevalence of depression and anxiety to be 49.5 and 38% vs. 48.9 and 34.7% among patients with asthma vs. patients with COPD, respectively. We also found that the prevalence of depression was nearly the same in both patients with asthma and COPD (49.5% vs. 48.9%), whereas the prevalence of anxiety was higher in patients with asthma than in patients with COPD (38% vs. 34.7%). Consistent with our results, a study (32) estimated that 70% of asthmatic patients have experienced some degree of anxiety. Symptoms of their condition and their fear of suffocation make asthma patients more likely to experience moderate levels of anxiety. This is contradicted by the findings of another study (33) that found mild depression in both asthma and COPD and that patient with asthma had moderate levels of anxiety while patients with COPD had high levels of anxiety.

For individuals with chronic respiratory conditions, the quality of their sleep is crucial to their physical and mental health (34). In the current study, individuals with COPD experienced poorer sleep quality more than those with asthma. This can be explained by the fact that COPD participants did not use sleeping aids in the previous month, and they experienced sleep disturbances “less than once a week.” This is consistent with a previous study (35) in which majority (94% of 51) of the patients with COPD reported “poor” sleep quality. According to a study (36), “getting up to use the bathroom” was the most frequent cause of sleep disruption. The multivariate regression analysis in our study showed that marital status (married), education level (pre-university level), and depression were significant predictors of PSQI among both asthmatic and COPD participants.

The PSQI sleep quality score and the HADS depression and anxiety scores were shown to have a high positive association in the current study. This supports a prior study’s finding that depression and sleep difficulty are related (37). Additionally, past research has indicated associations between sleep disruptions and both depression and anxiety levels in COPD patients, which is consistent with our findings (38).

Overall, the results of the current study indicate that depression, followed by anxiety and sleep disturbance, was the disorder that most affected people with COPD and asthma. According to the study’s findings, anxiety was more common in asthmatic participants than in COPD patients, whereas the incidence of depression was about the same in both groups (49.5 and 48.9%). According to earlier research, 10–57% of individuals with chronic respiratory illnesses have anxiety, and 10–59% experience depression (39, 40). Compared to patients with other chronic diseases, individuals with COPD had a higher risk of anxiety and depression, according to earlier research (41, 42).

The results of our study, which show a direct connection between anxiety and depression, are in line with earlier research. According to estimates from other studies, 26–43% of COPD patients also struggle with depression. Further research has revealed that COPD patients with depression are more likely to experience anxiety than COPD patients without depression (43, 44). Therefore, routine evaluations of individuals with CRDs’ mental health ought to take precedence.

The important features of the current study are the size of the sample and the inclusion of individuals with COPD and asthma who also had co-existing comorbidities, a novel strategy that was rare in earlier studies. However, there are certain limitations in this study. First, because the study was cross-sectional, no causality could be determined. Second, the subjectivity of the instruments used to collect data on sadness, anxiety, and sleep quality, because these data were self-reported and subjective. However, all individuals had recently received a COPD or asthma diagnosis from their doctors. Finally, we lack information on occupational status, which would add more information for future research. This study emphasizes the importance of routinely checking patients with asthma and COPD for poor sleep, depression, and anxiety, and it suggests developing therapies and/or management regimens improve their quality of life.

## 5. Conclusion

The study concluded that different degrees of sleep disturbance, anxiety, and depression are evident in people with asthma and COPD. While the frequency of depression was roughly the same in both groups, individuals with asthma had higher anxiety levels than those with COPD. It is important to properly evaluate the psychosocial needs of people with COPD and asthma. The mental health state of a person should be taken into account when treating an illness. According to this study, COPD, and asthma pose serious health risks, including reduced sleep quality, anxiety, and depression.

## Data availability statement

The original contributions presented in the study are included in the article/supplementary material, further inquiries can be directed to the corresponding author.

## Ethics statement

The studies involving human participants were reviewed and approved by The Research and Ethical Committee, Ministry of Health, authorized and approved the research proposal (approval number IRB KFHH no. H-05-HS-065). The patients/participants provided their written informed consent to participate in this study.

## Author contributions

The author confirms being the sole contributor of this work and has approved it for publication.

## Funding

The authors acknowledge the Deanship of Scientific Research, Vice Presidency for Graduate Studies and Scientific Research at King Faisal University, Al-Asha, for providing financial support under the Institutional Funding Committee (IFC) Ministry of Education, Saudi Arabia, “Grant no. INST202.”

## Conflict of interest

The author declares that the research was conducted in the absence of any commercial or financial relationships that could be construed as a potential conflict of interest.

## Publisher’s note

All claims expressed in this article are solely those of the authors and do not necessarily represent those of their affiliated organizations, or those of the publisher, the editors and the reviewers. Any product that may be evaluated in this article, or claim that may be made by its manufacturer, is not guaranteed or endorsed by the publisher.
